# Driving Change: Empowering Advocacy for Older Adults Through University Centers on Aging

**DOI:** 10.1093/geroni/igaf122.611

**Published:** 2025-12-31

**Authors:** Jodi Waterhouse

**Affiliations:** CU Anschutz Medical Campus, Aurora, Colorado, United States

## Abstract

This session will explore the pivotal role of University Centers on Aging in leading advocacy efforts that significantly impact older adult issues and the stakeholders who support their care. By building community consortiums to drive advocacy, education, and legislation, the CU Anschutz Multidisciplinary Center on Aging has successfully led, identified and addressed critical gaps. This session will provide a comprehensive, step-by-step guide to developing an effective advocacy strategy. Participants will gain insights from successful advocacy campaigns offering a blueprint for other states to establish similar working groups. Additionally, the session will cover strategies for securing state funding to support older adult issues, including positioning advocacy efforts within the Legislature to ensure the successful passage of legislative bills. Attendees will leave equipped with practical strategies to engage their state’s elected officials and drive support for Age-Friendly initiatives across the state and country.

